# A Pathogen Secreted Protein as a Detection Marker for Citrus Huanglongbing

**DOI:** 10.3389/fmicb.2017.02041

**Published:** 2017-10-23

**Authors:** Deborah Pagliaccia, Jinxia Shi, Zhiqian Pang, Eva Hawara, Kelley Clark, Shree P. Thapa, Agustina D. De Francesco, Jianfeng Liu, Thien-Toan Tran, Sohrab Bodaghi, Svetlana Y. Folimonova, Veronica Ancona, Ashok Mulchandani, Gitta Coaker, Nian Wang, Georgios Vidalakis, Wenbo Ma

**Affiliations:** ^1^Department of Microbiology and Plant Pathology, University of California, Riverside, Riverside, CA, United States; ^2^Center for Plant Cell Biology, University of California, Riverside, Riverside, CA, United States; ^3^Citrus Research and Education Center, University of Florida, Lake Alfred, FL, United States; ^4^Department of Plant Pathology, University of California, Davis, Davis, CA, United States; ^5^Department of Chemical and Environmental Engineering, University of California, Riverside, Riverside, CA, United States; ^6^Department of Plant Pathology, University of Florida, Gainesville, FL, United States; ^7^Texas A&M University – Kingsville Citrus Center, Weslaco, TX, United States

**Keywords:** citrus greening disease, HLB, effectors, disease diagnosis, antibody-based detection, bacterial secreted proteins

## Abstract

The citrus industry is facing an unprecedented crisis due to Huanglongbing (HLB, aka citrus greening disease), a bacterial disease associated with the pathogen *Candidatus* Liberibacter asiaticus (*C*Las) that affects all commercial varieties. Transmitted by the Asian citrus psyllid (ACP), *C*Las colonizes citrus phloem, leading to reduced yield and fruit quality, and eventually tree decline and death. Since adequate curative measures are not available, a key step in HLB management is to restrict the spread of the disease by identifying infected trees and removing them in a timely manner. However, uneven distribution of *C*Las cells in infected trees and the long latency for disease symptom development makes sampling of trees for *C*Las detection challenging. Here, we report that a *C*Las secreted protein can be used as a biomarker for detecting HLB infected citrus. Proteins secreted from *C*Las cells can presumably move along the phloem, beyond the site of ACP inoculation and *C*Las colonized plant cells, thereby increasing the chance of detecting infected trees. We generated a polyclonal antibody that effectively binds to the secreted protein and developed serological assays that can successfully detect *C*Las infection. This work demonstrates that antibody-based diagnosis using a *C*Las secreted protein as the detection marker for infected trees offers a high-throughput and economic approach that complements the approved quantitative polymerase chain reaction-based methods to enhance HLB management programs.

## Introduction

Huanglongbing (HLB) is currently the most destructive citrus disease that has caused tremendous damage to the citrus industry worldwide (Bové, 2006). HLB is believed to be associated with three *Candidatus* Liberibacter species: *Candidatus* Liberibacter asiaticus (*C*Las), *Candidatus* Liberibacter africanus (*C*Laf), and *Candidatus* Liberibacter americanus (*C*Lam) (Bové, 2006; Gottwald, 2010). *Ca*. Liberibacter spp. are Gram-negative bacteria belonging to the family Rhizobiaceae. In major citrus-growing areas including Asia, Brazil, and the United States, HLB is associated with *C*Las, which is the only HLB-associated species that has global distribution. *C*Las is mainly transmitted by the Asian citrus psyllid (ACP, *Diaphorina citri* Kuwayama) (Bové, 2006; Gottwald et al., 2007). During psyllid feeding and colonization of citrus trees, bacterial cells are introduced into the phloem and colonize phloem sieve tube elements (Bové, 2006; Gottwald, 2010). Typical disease symptoms include yellow shoots in tree sectors with thin canopy, branch dieback, and reduced fruit load. Leaves develop blotchy mottle with yellow islands, appearing in non-symmetric patterns in relation to the leaf midvein. Fruits are reduced in size, deformed (lopsided), often containing aborted seeds, and have uneven coloration (color inversion). Infected trees exhibit premature fruit drop, and eventually stop bearing fruits and decline (Bové, 2006; da Graça et al., 2015). Although some citrus species, such as limes and lemons, are relatively tolerant (Folimonova et al., 2009; Stover et al., 2010), and some resistance has been noted in citrus relatives (Ramadugu et al., 2016), all known citrus species and cultivars are affected by HLB. In addition to citrus, *C*Las can be experimentally transferred to periwinkle (*Catharanthus roseus*) as an alternative host. *C*Las replicates to high titers in the phloem of periwinkle (Tanaka et al., 2007) and infected leaves develop disease symptoms similar to HLB (Ding et al., 2015). Therefore, periwinkle has been used as a model for HLB studies (Garnier and Bové, 1983).

As curative HLB treatments for infected trees are still under development, the infected trees can serve as reservoirs for further pathogen dispersal via insect vectors (Schwarz and Van Vuuren, 1971; Zhang et al., 2011, 2014; Ehsani et al., 2013; Hoffman et al., 2013; Doud et al., 2014; Fan et al., 2014, 2016; Puttamuk et al., 2014; Al-Jumaili and Ehsani, 2015). Therefore, rapid and reliable diagnostic techniques that would allow accurate and timely identification of infected trees are an urgent need to establish effective HLB management. To date, robust HLB diagnosis remains challenging. The symptoms of HLB are easily confused with those caused by other diseases or nutrient deficiencies; moreover, the latency of HLB is highly variable, from a few months to a year or longer, depending on tree age, horticultural health, and other factors (Zhao, 1981; Shen et al., 2013). *In vitro* cultivation of the fastidious *Ca*. Liberibacter spp. has not been successful; as a consequence, traditional axenic culturing followed by microscopy and molecular and genetic analyses cannot be applied. Current detection of HLB infected trees relies mainly on polymerase chain reaction (PCR)-based assays targeting *C*Las DNA (Irey et al., 2006; Teixeira et al., 2008), which require the presence of the bacterial cells (or DNA) in the tested tissue for positive diagnosis. As a result, the outcome of PCR-based detection is greatly affected by the low titer and uneven distribution of the pathogen cells in infected trees (Ding et al., 2015). Furthermore, the ability of processing thousands of samples, necessary to track an epidemic, with PCR-based assays remains manpower and cost prohibitive (Gottwald, 2010; Rigano et al., 2014; Arredondo Valdés et al., 2016). Together, these challenges call for alternative methods for direct detection of *C*Las in citrus trees.

Serological assays detecting protein markers are widely used in disease diagnosis due to their high efficiency and low cost. Recently, a polyclonal antibody raised against the *C*Las OmpA protein was successfully used to detect HLB (Ding et al., 2015, 2016, 2017). OmpA is a major outer membrane protein of Gram-negative bacteria that is conserved in *Ca*. Liberibacter species. The anti-OmpA antibody was able to detect *C*Las cells in phloem tissues using a simple tissue imprint assay and could also be used to enrich *C*Las cells through immune capture to enhance PCR-based diagnosis (Ding et al., 2017). However, this antibody does not address the sporadic distribution of *C*Las cells in infected trees. Here, we pursued a different strategy and established the utility of a secreted protein as the marker to directly detect *C*Las in infected trees.

Bacteria possess protein secretion systems that are important for various cellular processes (Green and Mecsas, 2016). In particular, secreted proteins play an essential role in bacterial pathogenesis (Abramovitch et al., 2006; Sugio et al., 2011b). Genome analysis of *C*Las revealed the general Sec secretion system (Duan et al., 2009), which secretes proteins carrying an N-terminal signal peptide from the bacterial cells to the outside environment. Bioinformatic prediction combined with *Escherichia coli* alkaline phosphatase (PhoA) fusion-based experimental confirmation identified 86 proteins with functional Sec-dependent secretion signals from *C*Las (Prasad et al., 2016). Many of these proteins exhibited differential expression in citrus vs. in psyllids, indicating that they may function as “effectors” to manipulate host processes (Yan et al., 2013). Sec-delivered effectors (SDEs) are best studied in another insect-transmitted, phloem-colonizing bacterial pathogen phytoplasma (Hoshi et al., 2009; MacLean et al., 2011; Sugio et al., 2011a). The aster yellows phytoplasma strain (witches’ broom) is predicted to secrete 56 SDEs (Bai et al., 2009). Similar to *C*Las, phytoplasma cells are also restricted in the phloem of infected plants with limited movement; however, some SDEs have been shown to systematically move along phloem transport to root and shoot meristem and can also be uploaded from the phloem sieve cells to the neighboring tissues (Sugio et al., 2011b). In this way, these SDEs are not restricted to the sporadic bacterial infection sites, thereby could facilitate disease detection. Moreover, pathogen effectors are usually unique in specific species or even subspecies, making them promising candidates as detection markers with high specificity. A similar strategy was previously used to develop antibody-based detection methods for citrus stubborn disease (CSD), which is caused by the bacterial pathogen *Spiroplasma citri* (Shi et al., 2014). Similar to *C*Las, *S. citri* colonizes phloem tissue and exhibits uneven distribution in the infected trees. The antibody generated against a *S. citri*-specific SDE was able to detect CSD from samples collected from infected tree but showed negative diagnosis by PCR (Shi et al., 2014). These results encouraged us to implement the same concept to HLB.

Here, we report the utilization of *C*Las Sec-delivered effector 1 (SDE1) as a detection marker for HLB infected trees. SDE1 is conserved in *C*Las isolates and exhibits a relatively higher expression level in citrus than in psyllids. Using SDE1 as the antigen, we raised a polyclonal antibody and successfully detected *C*Las-infected citrus trees using western blotting, direct tissue blotting, vacuum-based dot blot immunoassay (DBIA), and enzyme-linked immunosorbent assay (ELISA). These serological methods are useful tools to improve HLB diagnosis.

## Materials and Methods

### Plant Materials and Sample Collection

Plant material was collected from healthy and *C*Las-infected sweet orange (*Citrus sinensis* L. Osb), mandarin (*C. reticulata* Blanco), grapefruit (*C. paradisi* Macf.), Mexican lime (*C. aurantifolia* Swingle), and pummelo (*C. maxima* Burm.) from the Citrus Research and Education Center (CREC), University of Florida (Lake Alfred, FL, United States), the Citrus Center, Texas A&M University-Kingsville (Weslaco, TX, United states), and the USDA-ARS Citrus Quarantine Facility in Beltsville, MD, United States.

Mature leaves and bark tissues were collected from 1-year-old branches of greenhouse raised plants as well as field trees in citrus groves. Citrus leaves with the blotching symptoms and confirmed for *C*Las infection by TaqMan qPCR were considered “symptomatic” samples; whereas leaves without typical HLB symptom but with *C*Las being detected by qPCR were considered as “asymptomatic” samples. Additional healthy samples collected from citrus trees maintained in the quarantine greenhouse of the Citrus Clonal Protection Program (CCPP) at the University of California, Riverside were also included as negative controls.

### Gene Expression Analysis of Candidate *SDE*s

Sec-translocon dependent extracytoplasmic protein analysis of the *C*Las was recently reported (Prasad et al., 2016). Here, we focused on the *SDE*s that are unique to *C*Las or *Ca.* Liberibacter spp. (**Table 1**) so that they could potentially be used as biomarkers for HLB. SDEs were predicted using signalP3.0 and signalP4.0 (Petersen et al., 2011). The *SDE1* genes in eight *C*Las strains were obtained from NCBI and their sequences were confirmed by PCR-sequencing. The sequence conservation of *SDE1* among the *C*Las strains was analyzed using clustalW (Thompson et al., 1994).

**Table 1:** Summary of Sec-delivered effectors predicted from *C*Las that were examined in this study.

Locus tag	MW (kDa)^a^	Predicted function	*D*-score^b^	Relative expression^c^	Signal peptide prediction		Homolog^d^	Reference
					SignalP3.0	SignalP4.0		
CLIBASIA_00460	9	Hypothetical protein	0.667	–	Y	Y	*C*Las-Psy62, *C*Las-ishi-1, and *C*Las-gxpsy	Prasad et al., 2016
CLIBASIA_03230	16	Hypothetical protein	0.705	3.52	Y	Y	*C*Las-Psy62, *C*Las-ishi-1, and *C*Las-gxpsy	Prasad et al., 2016
CLIBASIA_05315	14	Hypothetical protein	0.706	3.35	Y	Y	*C*Las-SGCA5, CLas-TX2351, *C*Las-ISHI, *C*Las-YCPsy, *C*Las-FL17, *C*Las-gxpsy, *C*Las-A4, *C*Las-Psy62	This study
CLIBASIA_05640	5	Hypothetical protein	0.668	–	Y	Y	*C*Las, *C*Laf, and *C*Lso-ZC1	Prasad et al., 2016

*^a^Molecular weight of mature proteins without the signal peptides. ^b^*D*-scores are the effective discrimination scores from SignalP4.0. Higher *D*-scores indicate greater probability of the candidate proteins as Sec-secreted proteins. ^c^Relative gene expression levels are presented as fold changes (log 2 ratio) in citrus vs. in psyllids. ^d^Homologous proteins identified by BlastP in the currently available *C*Las genome sequences as well as *C*Lso, which is the closely related *Ca*. Liberibacter solanacearum that associates with the potato zebra chip disease.*

Expression of potential *C*Las *SDE*s in HLB-infected citrus was determined by semi-quantitative RT-PCR. Total RNA was isolated from leaf and bark tissues using TRIzol^®^ (Invitrogen, United States) following the manufacturer’s instruction. Total RNA (1 μg) was treated with 1 U RNAse-free DNase I (Invitrogen, United States) and used for reverse transcription. The RNA concentrations were determined by spectrophotometer NanoDrop2000c (Thermo Fisher Scientific Inc., United States) and their integrity was assessed by agarose gel electrophoresis. The first-strand complementary DNA (cDNA) was synthesized in a 15 μL reaction, containing 12.5 μL (1 μg) RNA, 1 μL oligo dT_15-18_ primer (100 μM stock), and 1.5 μL dH_2_O. The mixture was incubated at 70°C for 10 min in a thermal cycler (MyCycler^TM^, Bio-Rad Laboratories, Inc.) and then immediately chilled on ice. The second strand was synthesized by adding 5 μL 5× First-Strand Buffer (250 mM Tris–HCl, 375 mM KCl, 15 mM MgCl_2_), 1.25 μL dNTPs (10 μM), 0.5 μL M-MLV reverse transcriptase (200 U/μL), 0.625 μL RiboLock RNase Inhibitor (40 U/μL), and 2.625 μL dH_2_O to the first strand reaction mix. The total mixture was incubated at 42°C for 1.5 h, then at 70°C for 15 min followed by immediate chilling on ice. PCR was carried out using gene-specific primer pairs (**Table 2**) for the *SDE* genes. The citrus *COX* gene was used as an internal control.

**Table 2:** Oligonucleotide sequences, annealing temperatures, and the predicted production sizes of PCR used in this study.

Target gene	Oligonucleotide sequences (5′–3′)	Size (bp)	*T*_m_ (°C)	Reference
CLIBASIA_00460-F	ATCCATTCGCCTCGTATT	181	50	This study
CLIBASIA_00460-R	GTTCACCTCCCATAAAATTATCT			
CLIBASIA_03230-F	TGACGGGAATCAGTATCACTTTCA	101	50	This study
CLIBASIA_03230-R	GCTAATGAACTTCAGAATAGCGATGT			
CLIBASIA_05315-F	ATACTCCGCGTGTTCCTGATGTCT	143	56	This study
CLIBASIA_05315-R	AGGAGGCGAAGCATGTGTTGAAGA			
CLIBASIA_05640-F	CTGAATCCTGATCAACTCTGTGAT	120	60	This study
CLIBASIA_05640-R	AGATAGTTTCGCACCCTTTGTAAG			
COX-F	GTATGCCACGTCGCATTCCAGA	70	56	Li et al., 2006
COX-R	GCCAAAACTGCTAAGGGCATT			
SDE1-F	AGGAAATATCGTGCGTAAAA	467	53	This study
SDE1-R	GCTCCAACATTTTTCTATGG			

Quantitative RT-PCR was used to determine the expression profiles of *SDE1* in different hosts (citrus, periwinkle, and psyllids) and at different disease stages of *C*Las-infected citrus tissues of Valencia sweet orange as described in Yan et al. (2013). Briefly, 15 fully expanded mature leaves (five leaves from each tree) from three symptomatic, three asymptomatic, and three healthy greenhouse raised citrus (5-year-old) were collected for RNA and DNA extraction. *C*Las titer was determined using the DNA extracts by TaqMan qPCR. The *SDE1* expression levels were determined by qRT-PCR. Relative transcript abundances were expressed as fold changes using DNA gyrase subunit A of *C*Las (*CLIBASIA_00325*) as the internal standard. All PCR reactions were performed in triplicate and the PCR products were separated by electrophoresis to confirm the presence of the products and their sizes. Data from duplicated experiments (expression in different host) were analyzed by one-way analysis of variance, followed by the all pairwise multiple comparison procedures (Tukey’s HSD test at *P* = 0.05). Data from duplicated experiments (expression at different disease stages) were analyzed by Student’s *t*-test at *P* < 0.01. All data were analyzed using SigmaPlot 13.0 statistical software package (Systat Software, Inc.). This experiment was repeated twice.

### Binding Affinity Analysis of the Polyclonal Antibody to SDE1

Direct binding of the antibody with SDE1 was evaluated using indirect ELISA. One hundred microliters of the antigen solution at different concentrations (20, 200, and 2,000 ng/mL) were used to coat ELISA plates (Immulon^®^ 2 HB Flat Bottom MicroTiter^®^ Plates, Thermo Fisher Scientific Inc., United States) by incubation at 4°C overnight. Wells were washed three times, 3 min each, using a HydroFlex^®^ microplate washer (Tecan, United States), with 300 μL of phosphate-buffered saline (PBS)-T buffer (PBS buffer containing 0.1% Tween-20). Plates were then blocked with 200 μL of 1× PBS containing 3%w/v non-fat milk at 37°C for 1 h. Wells were washed again as described above and incubated with 100 μL of anti-SDE1 antibody at different concentrations (5, 20, 100, and 1,000 ng/mL) at 37°C for 1 h. Plates were washed again and incubated with the goat-anti-rabbit IgG-horseradish peroxidase-conjugated secondary antibody (80 ng/mL, 1:5,000) for 1 h at room temperature. For signal detection, 100 μL of Ultra-3,3′,5,5′-tetramethyl benzidine TMB)-ELISA substrate solution (Thermo Fisher Scientific, Inc.) was added to each well and incubated in dark at room temperature until color development (up to 15 min). One hundred microliters of 2 M H_2_SO_4_ was added to stop the reaction, and the absorbance at 450 nm was measured using Tecan Plate Reader M200Pro. All samples were tested in triplicates.

### Detection of SDE1 in *C*Las-Infected Tissues Using the Anti-SDE1 Antibody

The binding specificity of the anti-SDE1 polyclonal antibody to SDE1 in citrus tissues was tested by western blotting. Healthy bark tissues were ground into fine powder in liquid nitrogen and then suspended in 1× PBS amended with 1× protease inhibitor cocktail (Sigma) at a ratio of 1:5 (i.e., 0.1 g tissue in 0.5 mL buffer). After being vortexed and incubated on ice for 30 min, supernatant was collected after a 20-min centrifugation at 13,000 × *g* at 4°C and then filtered through a 0.22 μm polyvinylidene difluoride (PVDF) syringe filter (EZFlow^®^). The tissue extract was spiked with purified SDE1 proteins and western blotting was used to examine specific binding of the anti-SDE1 antibody to SDE1 in citrus extracts. For the western blots, total proteins were separated by 12% SDS–PAGE. The concentrations of the primary and secondary antibody were 1:1,000 (200 ng/mL) and 1:3,000 (80 ng/mL), respectively.

Western blotting was also performed using *C*Las-infected tissue. Phloem-rich tissues (bark and midribs) were excised from symptomatic and asymptomatic citrus seedlings graft-inoculated with budwoods from the same citrus branch that was previously tested positive for *C*Las via TaqMan qPCR. The tissue powder was suspended in 2× Laemmli buffer (Laemmli, 1970) and western blotting was performed as described above. Tissues from a healthy seedling were used as the control.

### Direct Tissue Blot Immunoassay (DTBIA)

Young branches (1-year-old) were collected around the canopy of individual citrus trees. Stem tissues were cut with a steady motion to obtain a single plane cut surface using a sterile razor blade. The samples were “printed” by gently pressing the freshly cut cross-section of branches on nitrocellulose membranes (Plant Print Diagnostics S.L., Spain), leaving faint green-colored marks from the sap. The printed membranes were dried overnight at 4°C, then washed in TBS-T buffer (125 mM Tris–HCl pH = 7.4, 140 mM sodium chloride, and 3.0 mM potassium chloride, 0.05% Tween-20) at room temperature for 30 min to reduce non-specific binding. Membranes were blocked with 1× TBS-T containing 5% w/v non-fat milk at 4°C overnight and then incubated with the anti-SDE1 antibody (TBS-T containing 5% fat-free skim milk) and the antibody (200 ng/mL) in 1:1,000 dilutions, for 90 min at room temperature. The membranes were washed three times with TBS-T (5 min each time), and incubated with the secondary antibody (1:3,000 dilution, 80 ng/mL) for 1 h at room temperature. Signals were detected by SuperSignal^TM^ West Pico Chemiluminescent Substrate (Thermo Fisher Scientific Inc., United States) following the manufacturer’s instruction.

### Vacuum-Based Dot Blot Immunoassay (DBIA)

Stem samples from young branches were diced into small sections and ground to a fine powder using frozen stainless steel canisters in a stainless steel Kleco pulverizer (Kinetic Laboratory Equipment Company, Visalia, CA, United States). One gram of the tissue powder was suspended in 2 mL of extraction buffer containing 50 mM Tris–HCl, 150 nM NaCl, 1 mM EDTA, 1% Tween-20, 0.05% β-mercaptoethanol, and 10% glycerol. Each sample was vortexed for 5 s and incubated in ice for 30 min. Supernatants were obtained by two consecutive 25 min centrifugations at 13,800 × *g*. Fifty and 300 μL of the supernatants were then transferred to a clean tube and diluted 1:4 in carbonate coating buffer containing (100 mM sodium bicarbonate and 33.5 mM sodium carbonate, pH = 9.5). Diluted samples were applied in triplicates to a nitrocellulose membrane, pre-wetted in TBS-T buffer for 5–10 min, with the aid of a manifold apparatus (Schleicher & Schuell, Inc., Germany) under a vacuum. The spotted membranes were air-dried for 10 min at room temperature, and then processed as described in the DTBIA protocol except anti-SDE1 antibody concentration used was 1:400 (500 ng/mL).

### Indirect ELISA

Plant extracts were obtained as described above for the DBIA experiment. ELISA plates were coated with 250 μL of plant sample (1:4 dilutions in carbonate coating buffer) at 4°C overnight and then washed three times with the PBS-T buffer as described above. Each well was blocked with 200 μL PBS buffer containing 1% BSA and 0.1% Tween-20 and incubated at 37°C for 90 min. After blocking, plates were washed three times and incubated with 100 μL of anti-SDE1 antibody (200 ng/mL) for 1 h at 37°C. After three washes (5 min each time), the plate was incubated with 100 μL of secondary antibody (1: 1,500, 80 ng/mL) for 1 h at 37°C. Signals were detected as described above in the “binding affinity analysis of the polyclonal antibody” section. Data from duplicated experiments were analyzed by the Kruskal–Wallis one-way analysis of variance on rank, followed by the all pairwise multiple comparison procedures (Student–Newman–Keuls method; at *P* = 0.05). All data were analyzed using SigmaPlot 13.0 statistical software package (Systat Software, Inc.). This experiment was repeated twice.

### DNA Extraction and TaqMan qPCR

All the citrus samples that were examined by the anti-SDE1 antibody were also tested by qPCR to determine the *C*Las bacterial titer. Total nucleic acid was extracted from rich phloem tissues (bark) using a procedure optimized from a previously reported protocol for citrus (Osman et al., 2012). The system utilized Cryo-station and Geno Grinder 2010 (SPEX SamplePrep, Metuchen, NJ, United States), the MagMAX^TM^ Express-96 (Life Technologies, Carlsbad, CA, United States), and the MagMAX-96 Viral RNA Isolation Kit (Life Technologies, Carlsbad, CA, United States). Briefly, 250 mg of plant tissue was placed in an Eppendorf tube and submerged in liquid N_2_ for 30 s. Two 5/32″ stainless steel grinding balls were added in each tube along with 600 mL guanidine extraction buffer. The tubes were then placed into the cryo-blocks of Geno Grinder 2010 where the tissue was ground for 20–30 s at 1,750 *RPM.* The crude homogenate was centrifuged at 4°C for 30 min on a bench-top centrifuge at 14,000 *RPM* and the supernatant was then subjected to DNA extraction as described in Osman et al. (2012).

Two microliters of extracted DNA was used for quantitative TaqMan PCR (qPCR) with primers and probes described by Li et al. (2006) on a CFX96 Real-Time PCR System (Bio-Rad Laboratories, Inc.). Each reaction (20 μL) consisted of 250 nM (each) target primer (HLBas and HLBr), 150 nM target probe (HLBp), 300 nM (each) internal control primers (COXf and COXr), 150 nM internal control probe (COXp), and 2× iTaq Universal Probes Supermix (Bio-Rad Laboratories, Inc.). DNA extracted from healthy citrus tissues was used as the negative control.

## Results

### Expression Analysis of *SDE1*

**FIGURE 1:**
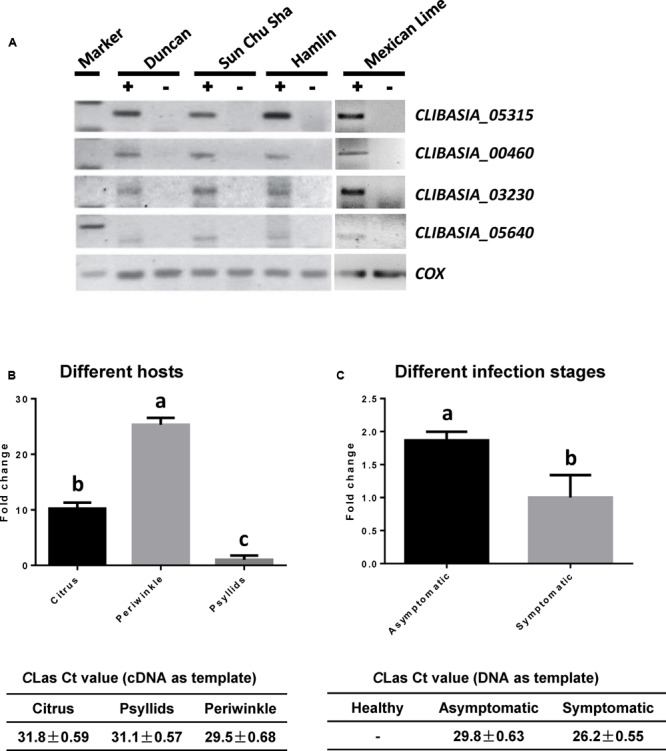
Expression profiling of predicted Sec-delivered effectors of *C*Las. **(A)** Semi-quantitative RT-PCR showing the expression of four *SDE*s in *C*Las-infected leaf tissues (+) of different citrus varieties. Healthy leaf tissues (–) from the same variety were used as the controls. **(B)** Quantitative RT-PCR showing the expression levels of *SDE1* in plant vs. insect hosts. Relative transcript abundances were determined using the gene encoding gyrase subunit A of *C*Las (*CLIBASIA_00325*) as an internal standard. The *C*Las titers in the plant [Valencia sweet orange (*Citrus sinensis*) and periwinkle] and insect tissues were determined by TaqMan qPCR. Different letters represent values that are significantly different (*P* < 0.05) according to one-way ANOVA with Tukey’s HSD test. Error bars represent the standard error of the mean (*n* = 5). **(C)** Quantitative RT-PCR showing the expression levels of *SDE1* in *C*Las-infected Valencia sweet oranges either showing HLB symptoms or remaining asymptomatic. *SDE1* is highly expressed in asymptomatic tissues despite relatively lower bacterial titer. The *C*Las titers in healthy, asymptomatic, and symptomatic tissues were determined by TaqMan qPCR. Different letters represent values that are significantly different according to Student’s *t*-test (*P* < 0.01). Error bars represent the standard error of the mean (*n* = 3).

Recent analysis on Sec-translocon dependent extracytoplasmic proteins of *C*Las (Prasad et al., 2016) revealed approximately 86 proteins that are potentially secreted by *C*Las through the Sec secretion system. We are interested in secreted proteins that are smaller than 20 kDa in size, potentially facilitating their movement in the infected trees along the photosynthate transport flow, and unique in *C*Las or *Ca*. Liberibacter so that they could be used as specific biomarkers for HLB detection. These genes were analyzed using semi-quantitative RT-PCR for their expression in *C*Las-infected tissues of citrus varieties with different HLB susceptibility levels, including Sun Chu Sha mandarin, Duncan grapefruit, Hamlin sweet orange, and Mexican lime. This analysis allowed us to narrow down to four *C*Las-specific SDEs (**Table 1**) from which we could detect expression in all four citrus varieties (**Figure 1A**).

**FIGURE 2:**
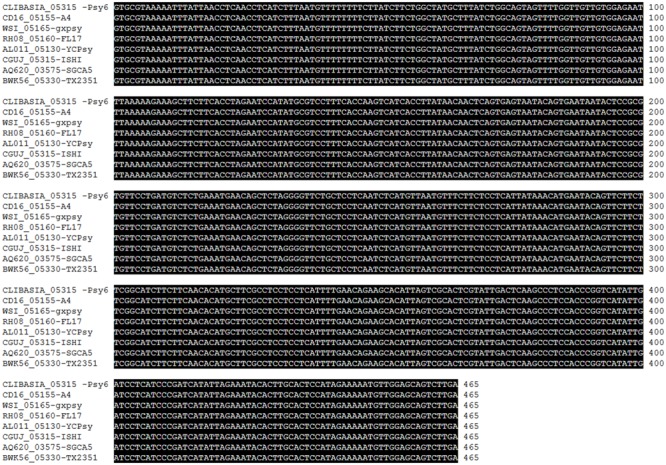
ClustalW alignment of the nucleotide sequences of *SDE1* from various *C*Las strains, including SGCA5 from California, TX2351 from Texas, ISHI from Japan, FL17 and Psy62 from Florida, and YCPsy, gxpsy, and A4 from China. *SDE1* sequences from these eight strains are 100% identical.

We were particularly interested in *CLIBASIA_05315* (hereafter *SDE1*), which showed the most consistent results in the semi-quantitative RT-PCR experiment. Further analysis on the expression profile of *SDE1* using quantitative RT-PCR shows that it is expressed approximately 10-fold higher in infected citrus tissues than in psyllids (**Figure 1B**). *SDE1* is also highly expressed in periwinkle (approximately 25-fold higher) compared to the expression level in the insect vector, suggesting that SDE1 proteins may accumulate to a high level in plant hosts. Furthermore, we were able to detect *SDE1* transcripts from the asymptomatic citrus seedlings, which had a lower bacterial titer. Intriguingly, the relative expression of *SDE1* (normalized by a housekeeping gene of *C*Las) was significantly higher (*P* = 0.01) in the asymptomatic citrus seedlings compared to that in the symptomatic trees (**Figure 1C**). These results indicate that *SDE1* is expressed at the relatively earlier infection stages, before the development of disease symptoms. Finally, *SDE1* is highly conserved among eight *C*Las strains whose genome sequences were available with 100% identity in nucleotide sequences (**Figure 2**). Taken together, these analyses support SDE1 as a promising biomarker for early HLB detection.

### Development of an SDE1-Binding Antibody

**FIGURE 3:**
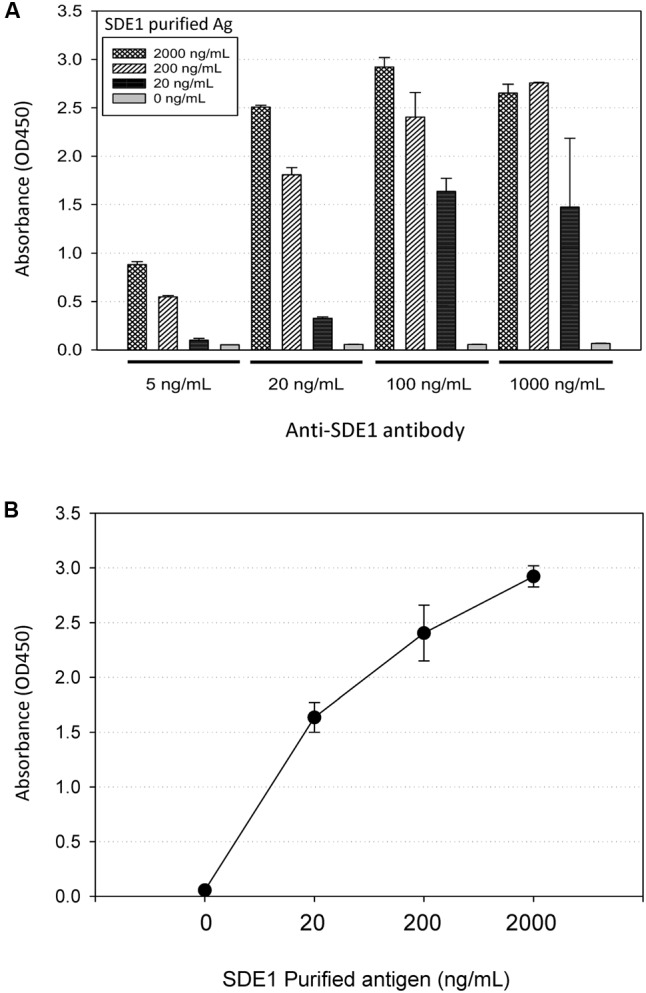
Anti-SDE1 antibody binds to SDE1 proteins efficiently. **(A)** Indirect ELISA showing dosage-dependent detection of SDE1 by the anti-SDE1 antibody. The purified SDE1 antigen (Ag) was coated on the ELISA plate at the concentrations of 0, 20, 200, and 2,000 ng/mL. The plate was incubated with the anti-SDE1 antibody at the concentrations of 5, 20, 100, and 1,000 ng/mL, and the signals were detected using TMB substrate solution. **(B)** A correlation between SDE1 concentrations and the absorbance values was at 450 nm. Graph shows data set plotted from the ELISA presented in **(A)** using 100 ng/mL of the anti-SDE1 antibody. Error bars represent standard error of the mean (*n* = 3).

To develop HLB detection using SDE1 as a biomarker, we raised polyclonal antibodies in rabbits injected with purified SDE1 protein as the antigen. A DNA fragment encoding the mature SDE1 protein (i.e., excluding the predicted N-terminal signal peptide, 1–24aa) was cloned into the *E. coli* expression vector pET28a. The recombinant protein was purified using nickel resins and used to inject rabbits for antibody production. The polyclonal antibody was purified from rabbit serum using affinity chromatography and evaluated for binding affinity to the SDE1 antigen using indirect ELISA. The binding affinity was tested using different concentrations of the purified anti-SDE1 antibody and SDE1 (**Figure 3A**). From this analysis, we determined that 100 ng/mL of purified anti-SDE1 antibody was the optimal concentration to use in indirect ELISAs and there was a positive correlation between the ELISA signal (absorbance at 450 nm) and the antigen concentration (**Figure 3B**). These analyses confirm that the anti-SDE1 polyclonal antibody has a high binding affinity to SDE1.

**FIGURE 4:**
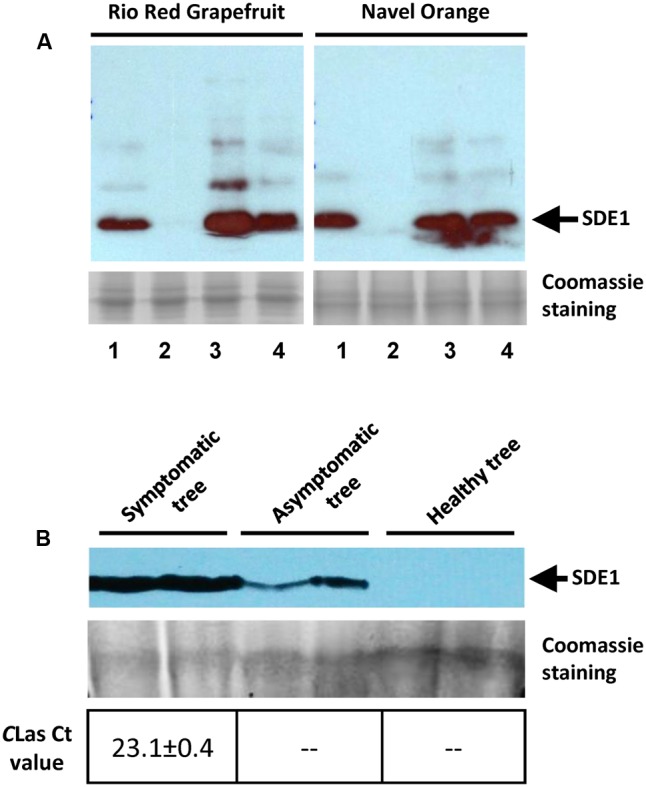
Anti-SDE1 antibody specifically binds to SDE1 in citrus extracts. **(A)** Western blots showing specific binding of the anti-SDE1 antibody to purified SDE1 proteins that were spiked into citrus extracts from healthy trees. Lane 1, SDE1 only (1 μg/mL); Lane 2, citrus extracts only; and Lanes 3 and 4, SDE1-spiked citrus extract with 5 and 1 μg/mL SDE1, respectively. The anti-SDE1 antibody was used at a concentration of 0.2 μg/mL. Healthy tissues were collected from citrus trees maintained at the quarantine greenhouse of University of California, Riverside. **(B)** Detection of SDE1 from *C*Las-inoculated or healthy citrus (Mexican lime) seedlings. Citrus seedlings were graft-inoculated with budwoods that were previously tested positive for *C*Las. Proteins extracted from symptomatic and asymptomatic seedlings were examined for the presence of SDE1 using western blotting. Bacterial titers in each sample were determined by qPCR. Only the HLB-symptomatic tree was confirmed as positive by qPCR. The arrows indicate the position of SDE1 based on its predicted molecular weight.

We next examined whether the antibody could detect SDE1 proteins in citrus extracts with high specificity. Purified SDE1 proteins were spiked into healthy citrus phloem extracts of Rio Red grapefruit and Navel orange, which were then detected by the anti-SDE1 antibody in western blotting experiments. Our results show that the healthy tissues had a minimal non-specific binding background, whereas the spiked samples showed a specific signal at a position consistent with the predicted molecular weight of SDE1 (14.3 kDa), in a dosage-dependent manner (**Figure 4A**). Furthermore, comparing with the purified SDE1 protein in PBS buffer, signals from the spiked sample in citrus extracts were even stronger, suggesting that citrus extracts do not interfere with antibody binding to SDE1.

We further detected SDE1 in *C*Las-infected citrus tissues using the anti-SDE1 antibody by western blotting. The same tissues were also subjected to TaqMan qPCR to determine the bacterial titer. Total proteins were extracted from bark tissues of healthy and *C*Las-infected Mexican lime seedlings. We were able to detect positive signals from protein extracts of the *C*Las-infected seedlings, no matter they showed HLB symptoms or remained asymptomatic, although the signals from the asymptomatic tissues were much weaker (**Figure 4B**). On the contrary, positive detection of *C*Las DNA by TaqMan qPCR was only achieved from the symptomatic tissue, but not from the asymptomatic tissue. These results suggest that: (1) SDE1 proteins accumulate during disease progression and/or with increasing bacterial titer; (2) SDE1 proteins may be present in citrus tissues independent of *C*Las cells; and (3) SDE1 is likely produced at an early infection stage. Taken together, these tests support further development of serological HLB detection methods using the anti-SDE1 antibody.

### Development of Antibody-Based HLB Detection Methods

**FIGURE 5:**
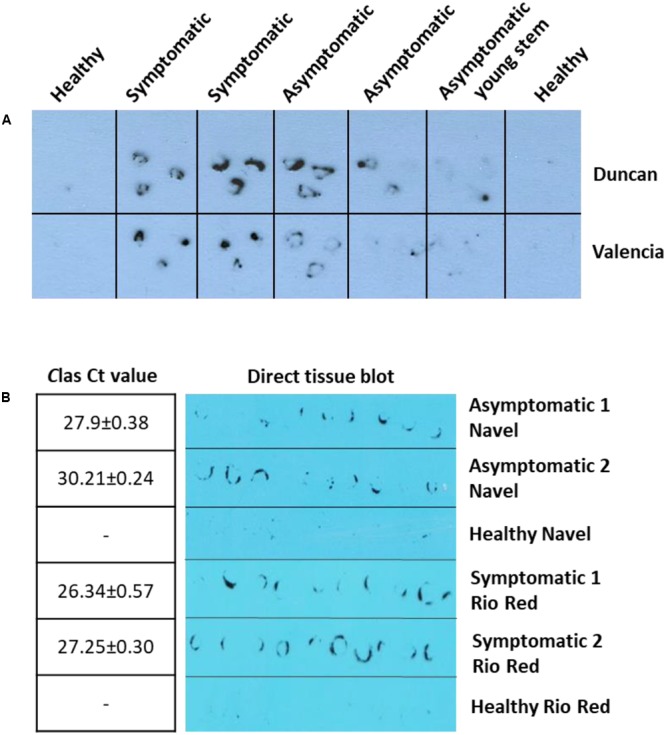
Anti-SDE1 antibody was able to detect *C*Las infection in greenhouse **(A)** and field **(B)** citrus trees using the direct tissue imprint assay. **(A)** Positive detection was achieved from both symptomatic and asymptomatic tissues of young stems from Valencia orange and Duncan grapefruit seedlings grown in the greenhouse. The fresh cuts of 1-year-old branches were printed on nitrocellulose membranes, which were then incubated with the anti-SDE1 antibody. **(B)** Positive signals, using the direct tissue imprint assay, were observed from both asymptomatic tissues of Navel orange and symptomatic Rio Red grapefruit sampled from the field. Bacterial titers were determined by TaqMan qPCR. All HLB-symptomatic and asymptomatic tissues were confirmed positive by qPCR (*n* = 3).

We next pursued the development of antibody-based detection methods for HLB using the anti-SDE1 antibody. First, we tried the DTBIA, which has been successfully used for the detection of citrus tristeza virus (CTV). The advantage of DTBIA lies in its effectiveness in field surveys due to simple equipment requirement and sample preparation (Lin et al., 1990; Garnsey et al., 1993; Knapp et al., 1995; Cambra et al., 1997; Amari et al., 2001). For this assay, we imprinted young branches (1-year-old) of citrus trees from grapefruits and sweet oranges on nitrocellulose membranes. The printed membranes were incubated with the anti-SDE1 antibody and the signals were detected by chemiluminescent substrates. The antibody was able to successfully detect *C*Las-infected trees from greenhouse (**Figure 5A**) and the field (**Figure 5B**). Consistent with the western blotting data shown in **Figure 4B**, positive signals were observed from both symptomatic and asymptomatic branches with weaker signals generated by the asymptomatic tissues, probably due to the lower bacterial titers (**Figure 5**). Furthermore, the signals were present exclusively in the regions corresponding to the phloem-rich tissues (the inner bark regions), where the bacterium and, presumably, the SDE1 protein should be located.

Although the DTBIA results suggest that sensitive detection of *C*Las infection was possible, the requirement of using the chemiluminescent substrates was inconvenient for diagnostic labs in non-research institutions due to the uses of X-ray films, a darkroom, and a film developer. Unfortunately, the less sensitive chromogenic substrates (e.g., TMB) were unable to generate convincing positive signals (data not shown). Since the targeted biomarker SDE1 is likely in a low abundance in early infected asymptomatic tissues, we further pursued the development of detection methods that would allow the uses of a larger amount of plant tissues/extracts in the tests so that the more convenient TMB substrate is sufficient to generate positive signals. For this purpose, we employed indirect ELISA and a vacuum-based DBIA. Compared to the DTBIA method, where only the plane of the cut surface of young branches is probed, ELISA allows up to 250 μL of plant extract (collected from 50 μg of plant tissue) that could be tested per reaction in a high throughput 96-well plate setup, and vacuum-based DBIA allows the application of plant extracts up to 1,500 μL collected from 300 μg of plant tissue.

**FIGURE 6:**
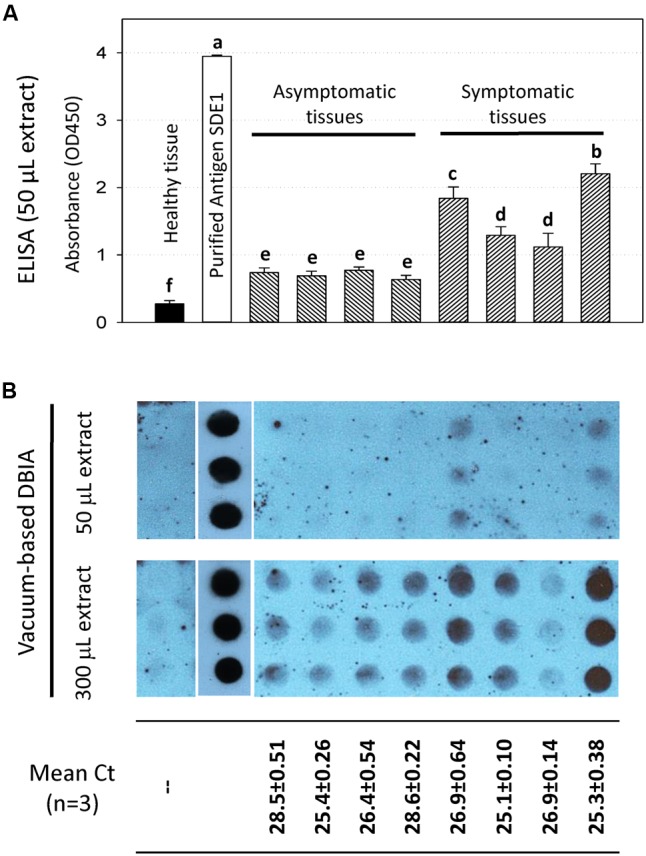
HLB detection using the anti-SDE1 antibody by ELISA **(A)** and vacuum-based dot blot assay **(B)**. **(A)** Indirect ELISA test showing quantitative detection of SDE1 in asymptomatic and symptomatic citrus tissues. ELISA plates were coated with plant extracts diluted in coating buffer and then incubated with the anti-SDE1 antibody. The asymptomatic and symptomatic tissues were collected from the same trees known to be *C*Las-infected. Different letters represent values that are significantly different at *P* < 0.05 according to the all pairwise multiple comparison procedures (Student–Newman–Keuls method). Error bars represent the standard error of the mean (*n* = 6). **(B)** Dot blots showing the detection of SDE1 from *C*Las-infected samples using the anti-SDE1 antibody. Different amount (50 or 300 μL) of citrus extracts were spotted on the nitrocellulose membranes with the aid of a manifold apparatus under vacuum. Stronger positive signals were obtained when a larger amount (i.e., 300 μL) of plant extract was used. Bacterial titers in the same tissues were determined by TaqMan qPCR. The same samples were examined by both assays.

Indeed, our results using ELISA (**Figure 6A**) and vacuum-based DBIA (**Figure 6B**) suggest that the sensitivity of *C*Las detection was increased. In the ELISA, protein extracts of asymptomatic and symptomatic tissues from four trees gave positive signals that are statistically significant (*P* < 0.001) compared to the healthy tissue (**Figure 6A**). In addition, stronger signals were observed from the vacuum-based DBIA when a larger amount of extract (300 μL vs. 50 μL) was used (**Figure 6B**). These assays suggest that SDE1 is a useful marker for HLB detection and the anti-SDE1 antibody is suitable for the development of serological diagnosis using multiple platforms.

## Discussion

Huanglongbing diagnosis with direct detection of the HLB-associated bacteria is challenging because of their uneven distribution and variable titer in the citrus trees. HLB diagnosis is also hindered by the extended latency periods for disease symptom development (Irey et al., 2006; Teixeira et al., 2008; Hu et al., 2013; Ding et al., 2015). On the other hand, direct detection of pathogen-associated molecules (i.e., biomarkers) allows for disease diagnosis with high specificity and has the potential to improve the timeline of detection before acute disease can manifest (Zhang et al., 2005).

The identification of biomarkers for early HLB diagnosis could play an important role in combating the disease. Combining multiple scientific fields using an interdisciplinary approach of genomic, transcriptomic, proteomic, and metabolomics analysis will provide a more complete understanding of the pathogen, its insect vector, and how it infects and interacts with a citrus tree. This knowledge is critical for the development of a comprehensive disease management strategy (Chin et al., 2014). Here, we report that the secreted protein SDE1 of *C*Las can be used as a detection marker for the identification of infected trees using various serological platforms. Detection methods using the anti-SDE1 antibody provide promising alternatives to the qPCR assays for *C*Las detection in a direct and specific manner by: (1) monitoring a different type of marker (i.e., proteins vs. DNA); (2) potentially increasing the chances of identifying infected trees, especially at the asymptomatic stage; and (3) allowing cost-efficient assays that support large-scale field surveys.

The advantage of using pathogen secreted proteins as biomarkers for HLB diagnosis lies in the *C*Las colonization of the phloem, which is the plant vascular system for photosynthate transportation. After secretion from the bacterial cells, SDEs could be dispersed along with the transportation flow, and therefore have an increased and relatively more even distribution in the infected trees. Although the actual distribution of *C*Las SDEs in citrus trees has not been determined, evidence from another phloem-colonizing bacterial pathogen, phytoplasma, strongly suggests that SDEs are able to systematically move in the phloem to reach the sink tissues (i.e., meristems) and also enter neighboring cells through plasmodesmata (Bai et al., 2009; Hoshi et al., 2009; Sugio et al., 2011a,b). As such, diagnostics based on SDEs represent a novel strategy that could better cope with the sporadic distribution of *C*Las cells in the citrus tree and the challenge of collecting the “right” tissue sample from a tree that contains *C*Las cells or DNA. Indeed, we have observed cases where asymptomatic tissues collected from *C*Las infected seedlings were tested negative by qPCR, but showed positive signals using the anti-SDE1 antibody. These observations are consistent with a wider distribution of SDE1 proteins compared to *C*Las cells in infected citrus trees.

Effectors are well-known as critical virulence factors that promote pathogen colonization and disease development (Bai et al., 2009; Hoshi et al., 2009; MacLean et al., 2011; Sugio et al., 2011a,b). Therefore, they are fast evolving during the co-evolutionary arms race with the hosts. As a result, effectors are usually highly variable in different pathogen species, or even subspecies, making them suitable for disease diagnosis with high specificity. SDE1 is unique to *C*Las; furthermore, it belongs to a “core” set of *C*Las SDEs that are produced in all isolates that have been tested. In addition to SDE1, *C*Las produces additional SDEs that could also be used as HLB diagnostic markers. Profiling on the expression of the complete SDE repertoire of *C*Las in common commercial citrus varieties, especially during early infection stages, will identify additional markers that could be incorporated into the current platforms to further enhance the sensitivity and accuracy of HLB diagnosis.

Compared to PCR, antibody-based detection assays are in general faster and more cost-efficient (Sankaran et al., 2010). Furthermore, various serological established platforms could be employed with different benefits. For example, the DTBIA platform is simple, rapid, and practical, suitable for large-scale field surveys (Garnsey et al., 1993; Knapp et al., 1995; Cambra et al., 1997; Amari et al., 2001). DTBIA does not require tissue processing, eliminating the need for even simple lab equipment such as homogenizers. In addition, vacuum-based DBIA can increase the chances of disease diagnosis when the targeted proteins are in low abundances in the tested samples (e.g., asymptomatic tissues), because it utilizes a larger sample volume which allows for higher antigen deposition onto the membrane. ELISA is the most widely used platform for serological diagnostics, allowing quantitative measurement and statistical analysis of target biomarkers. The use of 96-well plates is highly amendable for automation to increase throughput. Finally, new technologies have been developed and found applications in serological plant pathogen detection (Sharma and Sharma, 2016). For example, antibody-based nanosensors could be used to develop point-of-use devices for rapid and sensitive HLB diagnostics.

## Conclusion

Early HLB diagnosis remains a major goal for the citrus industry and regulatory agencies. Large-scale field surveys facilitate the identification of *C*Las infected trees, allowing growers to take appropriate actions toward disease management. We demonstrated that *C*Las secreted proteins can be serologically detected and used as biomarkers for HLB diagnosis. Although the presented assays showed promise as high-throughput and economic approaches for HLB diagnosis, additional validation, and evaluation, and most importantly, an optimized sampling protocol for a large number of samples (e.g., citrus varieties, tree ages, geographic locations, etc.) is required before this technology can be incorporated in the suite of HLB diagnostic tools.

## Author Contributions

DP, JS, ZP, EH, KC, ADF, JL, T-TT, SB, and ST did the experiments. DP, GV, and WM wrote the manuscript. NW, GC, VA, EH, KC, and ADF contributed to the writing. SF, VA, AM, NW, and GV provided materials, protocols, and intellectual insights. DP and WM designed the experiments.

## Conflict of Interest Statement

The authors declare that the research was conducted in the absence of any commercial or financial relationships that could be construed as a potential conflict of interest.
